# Proteomic studies of the abiotic stresses response in model moss – *Physcomitrella patens*

**DOI:** 10.3389/fpls.2012.00258

**Published:** 2012-11-22

**Authors:** Xiaoqin Wang, Yanli Liu, Pingfang Yang

**Affiliations:** ^1^Beijing University of AgricultureBeijing, China; ^2^College of Horticulture and Forestry Sciences, Huazhong Agricultural UniversityWuhan, China; ^3^Key Laboratory of Plant Germplasm Enhancement and Specialty Agriculture, Wuhan Botanical Garden, Chinese Academy of SciencesWuhan, China

**Keywords:** *Physcomitrella patens*, abiotic stress, proteomics

## Abstract

Moss species *Physcomitrella patens* has been used as a model system in plant science for several years, because it has a short life cycle and is easy to be handled. With the completion of its genome sequencing, more and more proteomic analyses were conducted to study the mechanisms of *P. patens* abiotic stress resistance. It can be concluded from these studies that abiotic stresses could lead to the repression of photosynthesis and enhancement of respiration in *P. patens*, although different stresses could also result in specific responses. Comparative analysis showed that the responses to drought and salinity were very similar to that of abscisic acid, while the response to cold was quite different from these three. Based on previous studies, it is proposed that sub-proteomic studies on organelles or protein modifications, as well as functional characterization of those candidate proteins identified from proteomic studies will help us to further understand the mechanisms of abiotic stress resistance in* P. patens*.

## INTRODUCTION

Being sessile, plants are continuously exposed to different biotic and abiotic stresses. The abiotic stress factors, which include drought, salinity, extreme temperature (cold and high temperature), heavy metal, and so on, are becoming more and more serious to the agricultural production all over the world. During the long evolutionary history, plants have evolved the ability to survive the adverse effects of stresses. Investigation of stress-responsive mechanisms has been a major topic for several decades in plant biology. Upon the reception of stress signal, plants could initiate a series of signal transduction, which triggers some protective responses to ensure a survival. Previously, it has been shown that stresses could result in the increasing of reactive oxygen species (ROS;  Allen et al., 2000), cytosolic Ca^2+^ concentration ( Apel and Hirt, 2004) and some other compounds that might function as secondary messengers and regulate downstream events such as protein phosphorylation and transportation ( Halfter et al., 2000). The whole signaling process will lead to changes in the expression of stress-responsive genes. In recent years, substantial progress has been made through the study of model plants, e.g., *Arabidopsis thaliana* and *Oryza sativa*. Many genes that play critical roles in abiotic stress response have been widely studied, especially some transcription factor encoding genes. Among the abiotic stress regulated genes, some are responsible for the biosynthesis of abscisic acid (ABA;  Rabbani et al., 2003). ABA is involved in many aspects of plant growth and development including the adaptation to abiotic stresses ( Wasilewska et al., 2008). Overlapping between the ABA and abiotic stresses signaling pathways indicate that the abiotic stress responses in plants are at least partially mediated by the ABA signal transduction ( Ishitani et al., 1997).

Evidence at the phylogenetic and paleobotanic aspects implies that the variety of terrestrial land plants evolved from a single colonization of the land about 480 million years ago (Kenrick and Crane, 1997). Except for the uncertainty of water supply, the land plants also had to confront with different environmental stresses, such as radiation and extreme temperature, when they first colonized a terrestrial habitat. The strategy for plant to deal with the terrestrial environment includes both the anatomical adaptation and the physiological and biochemical adjustments (Oliver et al., 2005). Most of the vascular plants have evolved some anatomical adaptation mechanisms. In contrast to the complex plants, the less complex plants have very simple anatomical structure. The stresses might directly work on individual cells in these plants. They adapt to environmental stress through biochemical adjustments at the cellular level. Based on the current knowledge, it is known that there are both common and species specific abiotic stress response mechanisms in plant kingdom. So it is necessary to study both complex and less complex plants in order to get a comprehensive idea about the abiotic stress response mechanisms.

Bryophytes, which comprise hornworts, mosses, and liverworts, are placed in a phylogenetic position between the green algae and the seed plants. It is believed that the ancestors of mosses and seed plants separated shortly after the transition from water to land at least 500 million years ago (Heckman et al., 2001; Hedges et al., 2004). They have very simple structures, which makes them ideal for studying the biochemical adjustment in response to abiotic stress. Recently, the moss species *Physcomitrella patens*has been getting more and more attention as a model plant for studying the unknown functions of genes as well as the response mechanisms to different abiotic stresses. This was mainly because of its characteristics which include simple cell structure and efficient homolog recombination (Schaefer and Zryd, 1997). In addition, the *P. patens*is the first bryophyte whose genome has been sequenced (Rensing et al., 2008). Its genome size is ~480 Mb, similar with that of the rice.

With the availability of genome information for different plant species, functional genomic approaches have been widely applied in the study of abiotic stresses resistance mechanisms in plants (Seki et al., 2001; Chen et al., 2002; Fowler and Thomashow, 2002; Kreps et al., 2002; Provart et al., 2003; Rabbani et al., 2003; Zeller et al., 2009). In the last two decades, proteomics has been shown to be a powerful tool in exploring many biological mechanisms at the systematic level. A lot of proteomic analyses have been conducted in *Arabidopsis*(Jiang et al., 2007) and rice (Yan et al., 2006; Li et al., 2010), which brought much deeper insight in the abiotic stress-responsive mechanisms. However, the proteomic studies in another model plant – *P. patens*are still very limited. In recent years, some proteomic studies including both profiling and phosphoproteomic analyses have been carried out in this moss species. Here, we summarized those studies in order to comprehensively understand the mechanisms of abiotic stress response in *P. patens* and get a clear idea for future work.

## COMMON RESPONSES AMONG DIFFERENT STRESSES

**FIGURE 1:**
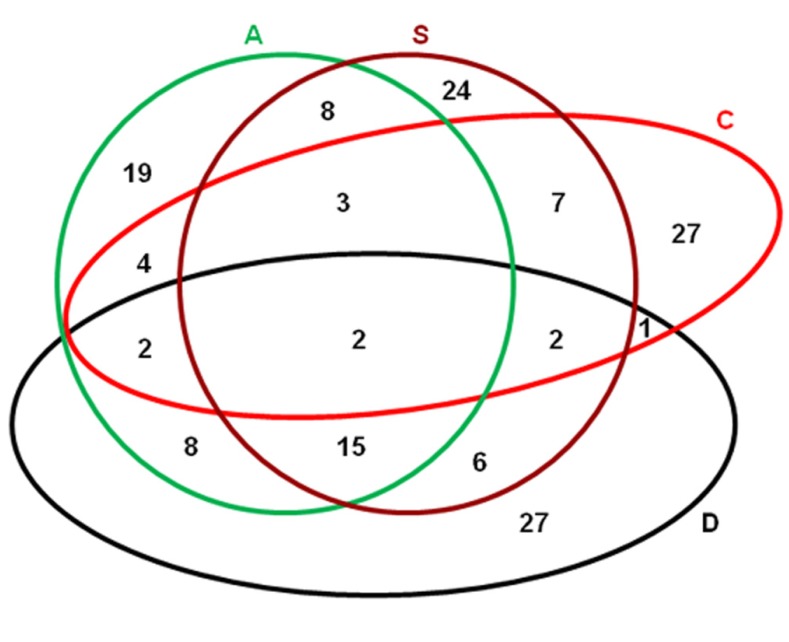
**Venn map showing the proteins that were regulated by different abiotic stresses and ABA treatments**. D, desiccation treatment: dehydrating to 90% fresh weight loss; S, salt treatment: >0.25 M NaCl for 3 days; C, cold treatment: 0°C incubation for >1 day; A, 50 µM ABA treat for 3 days. The raw data for this figure were from our previous studies ( Wang et al., 2008,  2009a,b, 2010).

As indicated previously, overlapping is a common phenomenon among the genes regulated by different abiotic stresses ( Ishitani et al., 1997). In our recent studies ( Wang et al., 2008,  2009a,b,  2010), when different abiotic stresses including dehydration, salt, cold, and ABA treatment were applied on *P. patens*, a lot of proteins were regulated coordinately. Comparative analyses showed that considerable overlapping existed among the proteins that were regulated by these four different stresses. Specifically, 2 were regulated by all four treatments; 12 were regulated by dehydration, salt, and ABA; 2 by dehydration, salt, and cold, 2 by dehydration, cold, and ABA; and 3 by salt, cold, and ABA. Thirty-four were regulated by two of the treatments (**Figure 1**; **Table 1**). Among all the differentially displayed proteins, 27, 24, 27, and 19 were regulated exclusively by dehydration, salt, cold, and ABA treatment, respectively (**Figure 1**). Based on these comparisons, it could be proposed that the relationship among the dehydration, salt, and ABA treatments is closer than that between anyone of these three with cold treatment, which has also been shown in rice and *Arabidopsis* at both transcriptomic ( Seki et al., 2002;  Rabbani et al., 2003) and proteomic levels ( Li et al., 2010). It has been suggested that ABA is involved in plants responses to environmental stresses, particularly drought and salinity ( Zhu, 2002). In *P. patens*, pretreatment with ABA could enhance its desiccation and freezing tolerance ( Khandelwal et al., 2010;  Richardt et al., 2010). The data in *P. patens* implies that the involvement of ABA in abiotic stress response might have been evolved prior to the divergence of moss and vascular plants during evolution.

**Table 1:** Proteins that were commonly regulated by cold (C), salt (S), dehydration (D), and ABA (A) ( Wang et al., 2008,  2009a,b, 2010).

Accession no.	Description	C	S	D	A
CAA51071.1	Glyceraldehydes 3-phosphate dehydrogenase	Y	Y	Y	Y
CAB85987.1	dnaK-type molecular chaperone hsc70.1	Y	Y	Y	Y
AAG61085.1	Intracellular pathogenesis-related protein like protein	Y	Y		Y
CAA68141.1	Chloroplast FtsH protease	Y	Y		Y
CAE47464.1	Lipoxygenase	Y	Y		Y
BAC85066.1	ATP synthase α subunit	Y	Y	Y	
NP_904216.1	ATP synthase CF1 α chain	Y	Y	Y	
AAQ88112.1	Actin	Y		Y	Y
BAC85044.1	Rubisco large subunit	Y		Y	Y
AAV65396.1	Physcomitrin		Y	Y	Y
AAY78603.1	PfkB-type carbohydrate kinase family protein		Y	Y	Y
BAC87878.1	Ribulose bisphosphate carboxylase small chain		Y	Y	Y
CAA05547.1	Heat shock protein 70		Y	Y	Y
CAA66484.2	2-Cys peroxiredoxin		Y	Y	Y
CAB75445.1	Fructokinase-like protein		Y	Y	Y
CAB43552.1	Phosphoribosyl diphosphate synthase		Y	Y	Y
CAB79338.1	HSP 70-like protein		Y	Y	Y
CAC43713.1	Unnamed protein product		Y	Y	Y
D84501	Hypothetical protein At2g12170		Y	Y	Y
F96795	Hypothetical protein F28O16.9		Y	Y	Y
Q9LKR3	Luminal binding protein precursor (BiP1) (AtBP1)		Y	Y	Y
BAA83481.1	Rubisco small subunit			Y	Y
BAC85045.1	ATP synthase β subunit		Y		Y
BAD08518.1	Light-harvesting chlorophyll *a*/*b*-binding protein	Y	Y		
BAD08519.1	Light-harvesting chlorophyll *a*/*b*-binding protein 2		Y	Y	
BAD94943.1	AMP deaminase like protein		Y		Y
CAA49506.1	Ketol acid reductoisomerase	Y		Y	
CAA49170.1	Nucleoside diphosphate kinase	Y	Y		
CAA67427.1	Thylakoid-bound ascorbate peroxidase			Y	Y
CAA73616.1	Multicatalytic endopeptidase		Y		Y
CAB87667.1	Subtilisin-like protease-like protein			Y	Y
CAB80488.1	Calcium-dependent protein kinase-like protein			Y	Y
CAB80784.1	AT4g00260		Y		Y
AAB20558.1	Light-regulated glutamine synthetase isoenzyme	Y	Y		
CAB87759.1	14-3-3-like protein GF14 ε	Y	Y		
CAB62598.1	*N*-hydroxycinnamoyl/benzoyltransferase-like protein		Y		Y
CAB54558.1	Plastid division protein ftsZ1			Y	Y
CAB52365.1	ATP synthase gamma chain		Y		
CAC03450	Ser/Thr-specific protein kinase-like protein			Y	Y
CAC42637.1	Unnamed protein product		Y	Y	
CAC43717.1	Unnamed protein product			Y	Y
CAC43712.1	Unnamed protein product			Y	Y
CAD38154.1	Putative ascorbate peroxidase			Y	Y
CAI84534.1	Unnamed protein product		Y		Y
C96608	Hypothetical protein F25p12.91			Y	Y
O65719	Heat shock cognate 70 kDa protein 3		Y		Y
O04005	Peroxiredoxin (thioredoxin peroxidase)			Y	Y
P22954	Heat shock cognate 70 kDa protein 2		Y		Y
Q39102	Heat shock cognate 70 kDa protein 1	Y	Y		
Q43127	Glutamine synthetase	Y	Y		
Q93Z66	Ribose-phosphate pyrophosphokinase 3		Y	Y	
Q9SYM5	Probable rhamnose biosynthetic enzyme 1		Y	Y	
Q39044	Vacuolar processing enzyme, β-isozyme precursor		Y		Y
1RP0A	Chain A, crystal structure of Thil protein		Y	Y	
1RP0B	Chain B, crystal structure of Thil protein		Y	Y	

Among all the stress-responsive proteins, the energy and metabolism related proteins were the largest group that was commonly regulated by different treatments. They accounted for ~29, 35, 41, and 29 of the total changed proteins in the treatment of salinity, dehydration, cold, and ABA, respectively. Generally, the anabolic proteins were decreased while the catabolic proteins were increased by the stresses ( Wang et al., 2008,  2009a, b,  2010). The expressional pattern of the proteins in this group indicated that the respiration was enhanced while the photosynthesis was repressed by the abiotic stresses, although most of the treatments (not including dehydration) did not result in the inhibition of *P. patens *growth ( Wang et al., 2008,  2009a,b,  2010). The repression of photosynthesis and enhancement of respiration seem to be a common response to the abiotic stresses in the plant kingdom. Further proteomics studies to the chloroplast and mitochondria might be very helpful to deeply understand the molecular mechanisms of *P. patens* resistance to different abiotic stresses.

In addition to similar changes of the energy and metabolism associated protein, some defense related proteins, such as APX, peroxiredoxin, and heat shock proteins (HSPs), were also changed in a similar way in responding to various abiotic stresses. Specifically, different members of HSP70s were dramatically changed upon the treatment of different abiotic stresses. Genomic analysis has shown the expansion of the HSP70 family to nine cytosolic members in *P. patens *( Rensing et al., 2008), whereas all algal genomes sequenced to date encode only one single cytosolic HSP70 ( van den Wijngaard et al., 2005). The expansion of some of the resistance related genes like *hsp70* during the evolution might help the plant to confront with a more complicated growth environment. Evolutionally, it seems that HSP70s are a crucial part of the plant apparatus ensuring resistance to various abiotic stresses.

## SPECIFIC RESPONSES TO EACH TREATMENT

Besides the common responses, *P. patens* also has specific responses to each individual abiotic stress.

### DEHYDRATION STRESS

To successfully colonize a terrestrial habitat, the plant should be able to adapt to the environment with an uncertain water supply. Most of the current land plants avoid harmful reductions in internal water supplies by using a variety of anatomical adaptation mechanisms, including root system, vascular tissues, and stomata, cuticles and lignin that restrict evaporative loss of water. However, the less complex plants, including the model moss species – *P. patens*, lack these kinds of anatomical adaptation, which means their primary response to dehydration is at the cellular level. And these cellular responses to water deficit have both economic and evolutionary importance that can affect the agriculture productivity and plant survival.

As mentioned, bryophytes are among the oldest terrestrial land plants. They should have the ability to survive severe dehydration stress. The ability to endure severe water deficit might commonly exist in bryophyte. It is reported that a bryophyte, *Tortula ruralis* is desiccation tolerant ( Oliver et al., 2004). Previous studies have shown that the *P. patens* plant could also recover its growth from a 92% water-loss ( Frank et al., 2005). But there is still controversy on this point since  Koster et al. (2010) reported that it could not survive water potential lower than -13 MPa (corresponding to 91% relative humidity). Pretreatment with ABA can increase *P. patens*’ desiccation tolerance and help it to survive a condition of 13% relative humidity ( Khandelwal et al., 2010;  Koster et al., 2010). Although it lacks the anatomical adaptations that commonly exist in the current dominant terrestrial plants, it could also adjust its cellular structure to avoid the severe damage of the water-loss. Besides the shrink of the cell and the dismantling of chloroplast inner membrane system, the cytoskeleton was also degraded during the drying process. All these features could be supported by the changes of related proteins, such as FtsZ, tubulin, and actin ( Wang et al., 2009b). Although the cell shrunk dramatically, the plasma membrane still kept integrated. It seems that the avoidance of plasma membrane damage is very important for plants to survive dehydration. Analysis of the membrane lipid constituents and its changes after the treatment of ABA might be helpful to understand the underlying mechanism.

Previous transcriptome analysis found an actinoporin-like proteins (ALPs) in drought stress ( Nishiyama et al., 2003). Recently,  Hoang et al. (2009) confirmed the up-regulation of this gene by dehydration and renamed it as bryoporin (*PpBP*). Besides dehydration, ABA, jasmonic acid (JA), salicylic acid (SA), and wound treatments could also up-regulated the expression of this gene. The PpBP protein contains hemolytic activity. Over-expression of this gene could enhance the dehydration tolerance of *P. patens*.

cDNA microarray analyses showed that a bunch of late embryogenesis abundant (LEA) proteins, mainly group 2 and 3, were induced upon the dehydration stress ( Oliver et al., 2004;  Cuming et al., 2007). This observation is consistent with the data in proteomic analysis. It is well known that the LEA proteins are very important for plant seeds experiencing the desiccation. They were also proved to play critical roles in plant vegetative tissues under drought or other abiotic stresses ( Cuming et al., 2007;  Hundertmark and Hincha, 2008). Microarray and proteomic analyses indicated that these proteins were mainly induced or up-regulated by dehydration treatment in *P. patens *( Hoang et al., 2009;  Wang et al., 2009b). This is also true in *Tortula ruralis* ( Oliver et al., 2004). Interestingly,  Saavedra et al. (2006) reported that the expression of dehydrin (group 2 LEA protein) encoding gene was up-regulated at both mRNA and protein level by different abiotic stresses including ABA, cold, salt, and dehydration in *P. patens*, which indicates that this protein might be also important in the response to these stresses. However, its function has only been confirmed under dehydration stress. Knockout of this gene could lead to *P. patens* lose the ability to recover from severe osmotic stress ( Saavedra et al., 2006). There still need more evidence to show when the expansion of LEA proteins’ function from dehydration stress to other abiotic stresses happened during the evolution.

### SALT STRESS

The* P. patens* has been shown to be high salt tolerant. The treatment of 300 mM NaCl did not result in any observable differences between the stressed and untreated plants ( Oliver et al., 2004;  Wang et al., 2008). It could even survive the treatment of NaCl with a concentration up to 350 mM ( Oliver et al., 2004).

Compared the differentially displayed proteins under the salt stress with those under other abiotic stresses, we could find that the proteins involved in the modulation of ionic and osmotic homeostasis were specifically regulated by the salt stress. It is well known that the ion absorption and compartmentalization are crucial for the normal growth of plants. High apoplastic levels of Na^+^ and C1^-^ could alter the aqueous and ionic thermodynamic equilibrium, and hence result in hyperosmotic stress, ionic imbalance, and toxicity. Thus, it is vital for the plant to re-establish its cellular ion homeostasis in high-salinity environments. During exposure to high levels of salinity, the maintenance of K^+^ and Na^+^ homeostasis is crucial, which depends on the proton-motive force created by the action of H^+^-ATPases and H^+^-pyrophosphatases ( Hasegawa et al., 2000). Phototropin and the 14-3-3 protein are primarily function as signaling pathway components ( Wu et al., 1997;  Takemiya et al., 2005). Recently, researchers have shown that these two proteins could work either cooperatively or independently to regulate the function of plasma membrane H^+^-ATPases and hence control the opening of stomatal and ion channels (e.g., the K^+^ channel;  Inoue et al., 2005;  van den Wijngaard et al., 2005). A PIIB-type Ca^2+^-ATPase, which involved in salt induced Ca^2+^ signaling is also essential for *P. patens*’ salt tolerance ( Qudeimat et al., 2008). In addition to these proteins, ABC transporters may also involved in the regulation of homeostasis, as it does in yeast ( Miyahara et al., 1996). Chloride can interfere with anionic sites involved in the binding of RNA and sugar-phosphates ( Serrano, 1996;  Hasegawa et al., 2000). Down-regulation of the chloride channel protein limits the transport of C1^-^ into cell. *P. patens* might be able to increase its salinity tolerance through constraining the transport of Na^+^ and Cl^-^. This phenomenon has also been reported in tomato ( Estan et al., 2005). Comparative analysis of the microarray data between *P. patens* and *Arabidopsis* showed that the functions of some other proteins such as DREB-like, Dof, and bHLH TAPs in salt response might be conserved during evolution ( Richardt et al., 2010).

### COLD STRESS

Previously, it has been shown that the *P. patens* could survive the treatment of -4°C ( Sun et al., 2007). The authors also showed that 0°C treatment could induce the *P. patens* to be resistant to a temperature as low as -7°C. These results indicate that 0°C treatment could initiate some biochemical or physiological processes that help *P. patens* to be more resistant to the freezing temperature. It is reported that pretreatment with ABA, NaCl and mannitol could increase the freezing tolerance of *P. patens *( Minami et al., 2003). In spite of this, the response of *P. patens* to cold treatment has little similarity with that to desiccation and salinity as we have mentioned above. One prominent characteristic of the cold response is that the expression of a series of transcription factors changed upon the treatment. The number of transcription factors is much more than those in any other treatment ( Wang et al., 2009a). It seems that there are more regulation happened at the transcription level in response to the cold stress. Quantitative real time (RT)-PCR data for some of the selected genes showed that the changes at the protein and RNA level are not always consistent.

### ABA TREATMENT

Plant hormones are important in regulating plant growth and development and its response to a variety of biotic and abiotic stresses. Significant progress has been made in identifying the key components involved in the signaling pathway of different plant hormones. Based on the current knowledge, ABA is known to play a crucial role in cellular responses to environmental stresses such as drought, cold, salt, wounding, UV radiation, and pathogen attack ( Rock, 2000). The ABA signaling pathway was found in *P. patens *( Knight et al., 1995), and some key components in this pathway, such as ABI3, ABI1 were also characterized ( Marella et al., 2006;  Komatsu et al., 2009). The study in *P. patens* will be useful to understand the evolution of ABA signaling pathway.

**FIGURE 2:**
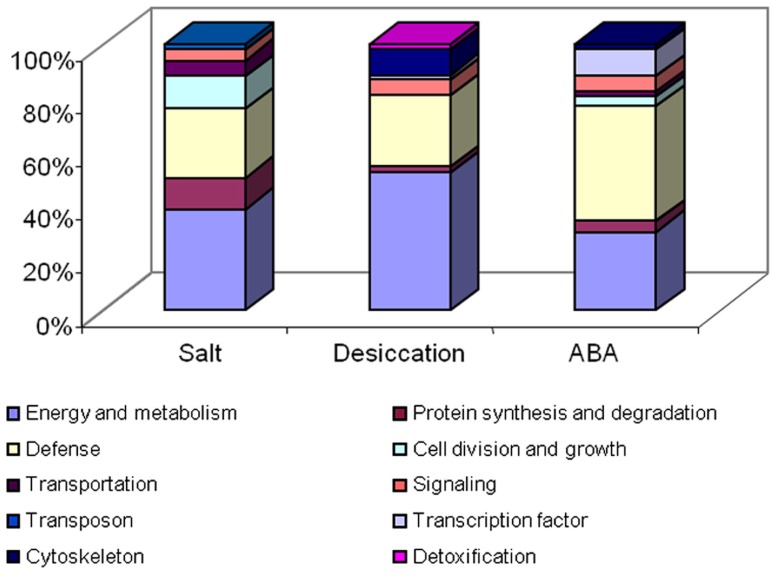
** Functional categorization of the differential displayed proteins under the treatment of salt, desiccation, and ABA **. This figure was based on our previous data ( Wang et al., 2008, 2009a,b, 2010).

The signaling pathways of dehydration and salinity were regarded as ABA dependent, which might help to explain the result that most of the proteins regulated by ABA were also regulated by the salinity and dehydration ( Wang et al., 2008,  2009b,  2010). Unexpectedly, microarray analysis showed that very little genes were commonly regulated by salt, dehydration, and ABA ( Saavedra et al., 2006). This implies that a systematic proteomic and transcriptomic analysis is necessary. In spite of this, the pattern of the protein changes in the ABA treatment is distinct from those in the dehydration and salt treatments ( Wang et al., 2008,  2009b,  2010). Proteomic analysis data showed that ABA treatment could regulate more defense related proteins and transcription factors than salinity and desiccation could (**Figure 2**). Specifically, some defense related proteins and transcription factors, such as receptor-like kinase, disease resistance proteins, lipoxygenase, and WRKY transcription factor 52, were exclusively up-regulated by ABA in our proteomic studies ( Wang et al., 2010). This may be explained by the fact that ABA involved in not only the dehydration and salinity signaling pathway but also the response to some other stresses. In addition to these proteins, we also found that some cell growth related proteins, such as expansin and extensin-like protein, were only regulated by ABA in all of the treatments. Expansin was known to have cell wall loosening activity and to be involved in the cell expansion and enlargement ( Cosgrove, 2000). Previously, it has been shown that expansin is linked to the action of auxin ( McQueen-Mason et al., 1992), gibberellin ( Cho and Kende, 1997), cytokinin ( Downes and Crowell, 1998), ethylene ( Cho and Cosgrove, 2002), and brassinosteroids ( Sun et al., 2005). Our proteomic data ( Wang et al., 2010) indicate that the expansin is also related to the function of plant hormone ABA at least in the very early terrestrial plant species. Prediction of the ABA responsive genes was conducted based on the *cis*-regulatory elements ( Timmerhaus et al., 2011), and over 90% of the predicted target genes were validated by microarray analysis ( Richardt et al., 2010). Taking these data together, we conclude that the ABA signaling pathway was largely conserved since the first land plants during the evolution.

## CONCLUSION

*Physcomitrella patens* has been a model system for decades because of its advantages in both genetic and structural aspects. Besides, *P. patens* has been proved to be highly resistant to different extreme abiotic stresses, such as high salinity, severe dehydration, and freezing temperature. With the availability of its genome information in the public database, exploring the stress resistance mechanism of this moss species at the proteomic level comes into reality. We have conducted a systematic proteomic analysis about its response to different abiotic stresses including high salinity, severe dehydration, cold, and ABA treatments. Except for the distinct responses to each treatment, common responses also exist. Comparison the responses between different treatments implies that there is a closer relationship between ABA and salt or ABA and dehydration than that between ABA and cold. Further functional analysis for those differentially displayed proteins under different stresses will help us to get more understanding to the stress resistance mechanisms in *P. patens* or even the whole plant kingdom.

As mentioned above, proteomic analyses have shown that a series of proteins involved in various cellular functions were regulated by different abiotic stress. To obtain further information about how these processes respond to stresses and how gene expressions were regulated, it might be necessary to initiate the sub-proteomic study in *P. patens*. To our understanding, this includes three major parts. First, it is about the proteomic analysis of organelles, such as nucleus, chloroplast, and mitochondria. Secondly, since the abiotic could result in ROS stress in the cell, and the accumulation of ROS in cell thereafter could lead to the carbonylation of the proteins, analysis of the protein carbonylation might be helpful to understand the protein turnover and functional alteration in response to stress. Thirdly, stress responses start with signal transduction. Currently, the knowledge of signaling in *P. patens* is still absent. Phosphoproteomics study might contribute a lot for us to understand the signaling pathway involved in stress responses. Furthermore, comparing the data acquired in *P. patens* with those from other model plants such as *Arabidopsis* and rice will bring us more general and comprehensive knowledge about the mechanisms of abiotic stress response in the plant kingdom.

## Conflict of Interest Statement

The authors declare that the research was conducted in the absence of any commercial or financial relationships that could be construed as a potential conflict of interest.
